# The positive climate impact of the Mediterranean diet and current divergence of Mediterranean countries towards less climate sustainable food consumption patterns

**DOI:** 10.1038/s41598-022-12916-9

**Published:** 2022-05-25

**Authors:** Simona Castaldi, Katarzyna Dembska, Marta Antonelli, Tashina Petersson, Maria Grazia Piccolo, Riccardo Valentini

**Affiliations:** 1 Department of Environmental, Biological and Pharmaceutical Science and Technology, University of Campania “Luigi Vanvitelli”, Via Vivaldi 43, 81100 Caserta, Italy; 2 Barilla Center for Food & Nutrition Foundation, Via Madre Teresa di Calcutta, 3/a, Parma, Italy; 3 Euro-Mediterranean Centre on Climate Change, Via Augusto Imperatore 16, 73100 Lecce, Italy; 4 Department for Innovation in Biological, Agro-Food and Forestry Systems, University of Tuscia, Via Camillo de Lellis 4, 01100 Viterbo, Italy; 5 Department of Ecology, Russian Timiryazev State Agrarian University, Timiryazevskaya st., 49, Moscow, Russia 127550

**Keywords:** Environmental impact, Climate-change mitigation

## Abstract

The Mediterranean diet (MD) is a world-renowned healthy dietary pattern. In the present study we analyse the climate sustainability of the MD and the greenhouse gas emissions (E_GHG_) associated with current dietary patterns in Mediterranean and non-Mediterranean EU countries, focusing on the major deviations from the MD health and environmental targets in Mediterranean countries. The E_GHG_ associated with dietary patterns were calculated for seven Mediterranean countries (Cyprus, Croatia, Greece, Italy, Portugal, Spain and Malta, referred to as 7MED) and the other 21 countries in the EU 28 (referred to as 21OTHER), using 2017 as the reference year. A new harmonised compilation of 3449 carbon footprint values of food commodities, based on a standardised methodology to extract information and assign optimal footprint values and uncertainties to food items, was used to estimate E_GHG_ of food consumption. Our findings show that the E_GHG_ associated with the ideal MD pattern, 2.3 kg CO_2_equivalents (CO_2_eq) capita^−1^ d^−1^, are in line with planetary GHG climate targets, though GHG emissions associated with food consumption in Mediterranean countries strongly diverged from the ideal MD. Both MED and 21OTHER countries were found to have comparable dietary associated E_GHG_ (4.46 and 4.03 kg CO_2_eq capita^−1^ d^−1^ respectively), almost double that expected from a sustainable dietary pattern. The primary factor of dietary divergence in 7MED countries was found to be meat overconsumption, which contributed to 60% of the E_GHG_ daily excess (1.8 kg of CO_2_eq capita^−1^ d^−1^).

## Introduction

In the present and future development of humankind, healthy food systems have an important role to play in both contributing to keeping greenhouse gas emissions (E_GHG_) within planetary limits of sustainability and in improving health outcomes^1,2^. In terms of the former, feeding the global population currently accounts for 21–37% of total net anthropogenic E_GHG_^1^. In terms of the latter, non-communicable diseases, such as diabetes, cardiovascular diseases, cancer and chronic respiratory diseases, are responsible for 41 million deaths each year, and one in five deaths are attributed to unhealthy diets globally^3^.

A dietary shift towards more sustainable food systems would be an effective measure to contribute to global E_GHG_ reduction targets^1,2^ and is a key element of the European Union’s new Farm to Fork strategy, a pivotal component of the European Green Deal’s ambition to decarbonise the continent by 2050. Tackling health and sustainable food consumption patterns is also key to improving the nutritional status and health of EU citizens^4^. In this respect, the Mediterranean diet (MD) could play an important role in EU climate targets, as it is recognized as a healthy dietary pattern^5–8^ that contributes to environmental^7,9^ social and cultural services^10,11^. The MD^10^ is, in fact, rich in plant-based foods (cereals, fruits, vegetables, legumes, tree nuts, seeds and olives) with moderate-to-high consumption of fish and seafood, moderate consumption of eggs, poultry and dairy products (cheese, milk and yoghurt) and low consumption of red meat, with extra-virgin olive oil used as the principal source of added fat. The MD is still evolving, and there are many versions expressing the different food cultures of the Mediterranean region^6^. In 2010, the MD was recognised by UNESCO^12^ as an intangible cultural heritage of humanity, raising its status to a model which should be fostered in the agricultural, political, cultural, economic and public health contexts of the Mediterranean area.

A recent global analysis of food-based dietary guidelines (FBDG)^8^ demonstrated that a core set of dietary guidance beneficial to health appears “nearly universally across countries” and in line with WHO global guidance; nonetheless, for many FBDGs, further improvements could be achieved towards greater health benefits^7,8^. Furthermore, from an environmental perspective, it was found that the national-level reductions in environmental impacts achieved through the adoption of FBDGs were mostly moderate, and most FBDGs were not in line with global environmental targets, especially E_GHG_ reduction targets^7^.

The present study evaluates the adherence of the MD to global E_GHG_ reduction targets and investigates the current food consumption patterns and associated E_GHG_ in the EU, focusing on Mediterranean countries to evidence major deviations from MD health and environmental targets.

For this purpose, we calculated the average dietary consumption pattern, using 2017 as a reference year, for two groups of countries. The first group (7MED) includes the six European Mediterranean pioneer countries on which the first studies of the MD were based^10^, Cyprus, Croatia, Greece, Italy, Portugal, Spain, plus Malta. The second group (21OTHER) includes the other 21 countries of the EU 28 in 2017. E_GHG_ associated with the analysed dietary patterns were based on the carbon footprint (CF) concept^13^, which uses a life cycle approach to incorporate E_GHG_ from land management, industrial processes, transport and energy to cover all the possible E_GHG_ associated with the functional unit of a food product. The current estimates of dietary consumption and associated E_GHG_ are mainly based on the CF of fresh and minimally processed products and exclude beverages.

## Materials and methods

### Carbon footprint values of food commodities

The CF data, used in this study to estimate E_GHG_ associated with food, were derived from a harmonised compilation of CF footprint values of food commodities, the Su-Eatable LIFE (SEL) database, published by Petersson et al. 2021^14,15^. The SEL database^14^ was created using a standardised methodology to extract information from published CF studies and to assign CF values and uncertainties to food items^15^. The database contains 3349 CF values (with Eurocentric prevalence of 50–60%), extrapolated from 841 publications (1998–2019) and distributed in four main commodity groups: fresh or minimally processed (frozen, canned or dried) plant-based food commodities (978 CF data), plant-based industrially processed food (457 CF data), fresh or processed food commodities derived from terrestrial animals (1496 CF data) and food commodities from the ocean and freshwaters (263 CF data). Data from single studies, once harmonized to refer to the same system boundaries and functional unit, are further aggregated to represent the food items commonly found in the market definitions (apple, yogurt, potato, etc.). Each food item is categorised by typologies (a group of items with similar characteristics) and within some typology category, by sub-typologies (e.g. the typology “shellfish” includes the following sub-typologies: crustacean, bivalves and cephalopods). The CF database comprises a total of 85 food typologies, 11 food sub-typologies and 323 food items^14,15^. A robust methodology of uncertainty attribution, based on three statistic indicators, is applied to define the statistically representative CF value of each food commodity (details of the statistical approach are fully explained in Petersson et al.^15^).

### Daily per capita E_GHG_ associated with food consumption of EU citizens

We estimated the annual per capita E_GHG_ associated with food consumption of EU citizens, differentiating 7MED and 21OTHER, using the following procedure.

Plant based food, meat and fish consumption data were extracted from the FAOSTAT food balance database^16^ by selecting the element “food” (code 5142), the reference year 2017, the 28 countries part of the European Union in 2017 and FAOSTAT items codes reported in Supplementary Table 1. As CF of fresh and hard or semi-hard cheese differ significantly (Supplementary Table 2), consumption data for these two food groups were calculated separately based on data reported in CLAL.it^17^ (Supplementary Table 3). Dairy consumption was calculated as production plus import minus export, assuming negligible storage of dairy products from the previous year. CLAL.it data of dairy production is based on EUROSTAT data from NewCronos (apro_mk_pobta and apro_mk_farm)^18^, while import/export data reported by CLAL.it are derived from IHS/GTA data (see Supplementary Table 3).

The aforementioned food consumption data were aggregated into 20 food groups, considered as the most representative in the average dietary definitions: cereals, starchy tubers, vegetables, fruit, pulses, nuts and seeds, sweeteners, other vegetable oils, olive oil, butter, milk, cream, yogurt, cheese, beef, mutton and goat, pork, poultry, eggs, fish and shellfish.

The two country groups 7MED and 21OTHERS were analysed separately and, for each of them, the *annual weighted per capita E*_*GHG*_* of each food group (E*_*FG*_*)* (kg CO_2_eq capita^−1^ yr^−1^) was calculated with Eq. 1:

1 $$E_{FG} = \mathop \sum \limits_{1}^{n} \left( {\frac{{item_{i} \left( {g_{1,2} } \right)}}{{Population \left( {g_{1,2} } \right)}} \times CF_{i} } \right)$$

where *item*_*i*_ (*g*_1,2_) represents the total kilograms of the *i-*th* item* belonging to the food group *FG* (including *n* items) consumed by all the citizens of either group in 2017; *Population *(*g*_1,2_) is the total number of individuals present in either groups in 2017; *CFi* is the CF value (kg CO_2_eq kg^−1^ product) attributed to the *i-*th* item* extracted from the SEL database^14^ (data used for calculations are reported in Supplementary Table 1).

The daily per capita CO_2_eq emissions (E_GHG_) estimated for the average citizen of 7MED or 21OTHERS were calculated as the sum of the emissions of the 20 food groups divided by 365 (days in a year) with Eq. 2:

2 $$Daily\; per\; capita\; E_{GHG} = \frac{{\mathop \sum \nolimits_{1}^{n} \left( {E_{FG} } \right)_{i} }}{365}$$

### Daily per capita E_GHG_ associated with the Mediterranean diet

To determine which are the food groups characteristic of the MD pattern and their weekly consumption frequency, we relied upon the comprehensive literature review of the MD by Davis et al.^19^ and on the MD pyramid guidelines^10^. The reference documents report the minimum–maximum limits of food group consumption frequency over a week but do not specifically recommend serving size^10,19^, as the exact make-up of a diversified, balanced and healthy diet will vary depending on individual characteristics (e.g. age, gender, lifestyle and degree of physical activity), cultural context, locally available foods and dietary customs^20^. To define the impacts of a reference MD dietary plan, three different weekly dietary scenarios (Table 1) were created where the daily intake of plant-based food groups (cereals, vegetables, fruit, etc.) was left constant in the three scenarios, while the consumption frequency of animal protein foods (poultry, red meat, fish, eggs, and dairy) was varied within the limits imposed by the MD guidelines. Portions (g) and the intake frequency of food groups in each of the three scenarios were calibrated within the MD limits on the following bases: (a) the average energy requirement by an average EU citizen (about 2380 kcal capita^−1^ day^−1^), estimated by the EFSA Panel on Dietetic Products, Nutrition and Allergies^21^, which takes into account different age and sex groups and various levels of physical activity associated with sustainable lifestyles in healthy individuals; the energy requirements were distributed over three main meals, breakfast, lunch and dinner plus two snacks; (b) the content of the main nutritional elements and caloric values (kcal) of each food group^22,23^; (c) healthy diet standards set by the World Health Organization^20^ and by the Harvard T.H. Chan School of Public Health’s Healthy Eating Plate^24^. The WHO recommends a healthy diet to include fruit, vegetables, legumes, nuts and whole grains, at least 400 g of fruit and vegetables per day, less than 10% of total energy intake from free sugars, less than 30% of total energy intake from fats, minimal to no trans fats and less than 5 g of salt per day. The Healthy Eating Plate provides a guide on balanced meals and divides an ideal plate into four parts: half of the plate for vegetables and fruits, a quarter for whole grains and the last quarter for healthy proteins.

**Table 1:** Mediterranean dietary pattern scenarios used to define the MD reference diet in this study.

		Scenario 1				Scenario 2				Scenario 3			
Food type	MPD frequency indications	Daily portion (g)	Weekly frequ	Weekly input (g)	CF kg CO_2_eq kg^−1^	Daily portion (g)	Weekly frequ	Weekly input (g)	CF kg CO_2_eq kg^−1^	Daily portion (g)	Weekly frequ	Weekly input (g)	CF kg CO_2_eq kg^−1^
Cereals	1–2/meal	300	7	1900^a^	1.19^d^	300	7	1900^a^	1.19^d^	300	7	1900^a^	1.19^d^
Legumes	> 2/week	50	**3**	150	0.49	50	**4**	150	0.49	50	**2**	100	0.49
Potatoes	< 3/week	300	2	600	0.24	300	2	600	0.24	300	2	600	0.24
Vegetables	> 2/meal	400	7	2800	0.41	400	7	2800	0.41	400	7	2800	0.41
Fruit	1–2/meal	300	7	2100	0.45	600	7	4200	0.45	450	7	3150	0.45
Nuts&seeds	nuts 1–2/day	30	7	210	1.10	30	7	210	1.10	30	7	210	1.10
Red meat^§^	< 2/week	100	**1**	100	18.09^b^	100	**1**	100	18.09^b^	100	**1**	100	18.09^b^
Poultry	2/week	100	**2**	200	3.88	100	**2**	200	3.88	100	**3**	300	3.88
Cheese	NS	83.3^c^	**3**	250	4.38^c^	75^4^	**1**	75	6.83	83,3^c^	**3**	250	4.38^c^
Dairy^§§^	2/day	300	7	2100	2.05	150	7	1050	2.05	300	7	2100	2.05
Butter	0	0	0	0	8.48	0	0	0	8.48	0	0	0	8.48
Eggs	2–4/week	100	**2**	200	3.20	100	**2**	200	3.20	100	**3**	200	3.20
Vegetable oil^§§§§^	every meal	40	7	280	3.27	40	7	280	3.27	40	7	280	3.27
Fish	> 2/week	150	**3**	450	4.52	150	**4**	600	4.52	150	**2**	300	4.52
Sugar equ.^§§§^	NS	20	7	140	0.82	20	7	140	0.82	20	7	140	0.82

Three patterns of MD differing for weekly frequency distribution of protein sources, within the recommended limits provided by the Mediterranean pyramid diet (MPD)^10,19^ and complying with nutritional indications^20–24^. Portions and weekly frequency have been calibrated for an average EU citizen energy requirement^21^. The carbon footprint (CF) values reported in this table represent weighted CFs based on the relative proportion of each food item present in each specific group (see methods for more details). Single food item CF values were extracted from the SU-Eatable LIFE database^14,15^.

^§^Red meat is the sum of beef meat, mutton and goat meat, and pork meat, §§milk and white yogurt, §§§sugar equivalents in jam or honey, §§§§Olive oil is the fat source suggested by MDP.

^a^2 portions per week of pasta or bread are substituted with 2 portion of potatoes, considering an equivalent ratio bread: potato of 1: 3 (g fresh weight).

^b^CF of red meat is the average of the bone free meat CF for beef meat (25.75 kg CO_2_eq kg^−1^), pork meat (5.72 kg CO_2_eq kg^−1^), mutton and goat meat (25.23 kg CO_2_eq kg^−1^).

^c^3 portions of cheese considered as 2 portions (100 g each) fresh cheese and 1 portion 50 g hard/semi hard cheese, the weighted CF is calculated accordingly starting from a value of CF of 5.45 kg CO_2_eq kg^−1^ for fresh cheese and 9.59 kg CO_2_eq kg^−1^ for semi hard/hard cheese.

^d^1 portion of cheese considered as the average between 1 portion (100 g) fresh cheese and 1 portion (50 g) of hard/semi hard cheese alternate through the weeks or as eating half portions of both cheese typologies in the same week (50 g fresh and 25 g hard/semi-hard); the CF weighted value is calculated accordingly.

Numbers in bold underline the frequency of different protein sources within each of the 3 dietary scenarios.

Next, food items used for the estimates of consumption data, were aggregated into 15 food groups (Table 1), based on MD Pyramid guidelines^10,25^ and a CF value (functional unit kg CO2eq. kg^−1^ or l^−1^ food) was assigned to each food group in each dietary scenario (Table 1). For this specific task, the CF value attributed to each food group was estimated as a weighted CF value (*weighted CF*_*FG*_) using the CF values extracted from the SEL database^14^. This approach avoids wrongly attributing equal environmental “weight” to items that only have a moderate level of consumption by EU citizens within a food group. The following Eq. (3) was used:

3 $$Weighted \, CF_{FG} = \mathop \sum \limits_{1}^{n} \left( {CF_{i} \times freq._{i} } \right)$$

where *CF*_*i*_ is the CF value of the *i-*th food item of the food group FG (including *n* items); *freq.*_*i*_ is the relative frequency of consumption of the *i-*th food item in the food group FG (food groups are reported in Table 1). For the specific case of the item “wheat”, which is in the food group “cereals”, an average CF value was calculated from bread, flour and pasta CFs extracted from the database by Petersson et al.^19^. For the specific case of the *weighted CF*_*FG*_ of the food group “cheese*”*, two items were considered separately, fresh cheese and semi-hard/hard cheese, due to their different caloric value and nutrient density and different CFs^14,15^ (Table 1).

To estimate the total E_GHG_ associated with each of the three scenarios of the MD (Table 1), the total weekly intake (g) of each food group was multiplied by its corresponding weighted CF_FG_; the total weekly emissions of each food group were then summed and then divided by 7 (days in a week) to express the result as average per capita daily emissions.

### Deviations of food consumption patterns from the Mediterranean diet in Mediterranean countries

To estimate the deviation of the current daily consumption of 7MED from the ideal MD pattern, the *weekly consumption rate of the average Mediterranean citizen* (WC_FG_) (grams capita^−1^ week^−1^) was calculated for each food group reported in Table 1 with Eq. 4:

4 $$WC_{FG} = \mathop \sum \limits_{1}^{n} \frac{{item_{i} }}{Population } \times \frac{7}{365}$$

where *item*_*i*_ represents the total kilograms of the i-th item, belonging to the food group FG (including *n* items), consumed by all 7MED citizens in 2017; *Population* is the total number of individuals in the 7MED countries in 2017.

For this specific analysis, to compare the dietary intake to the correct food consumption term “bone free meat”, the average bone free meat consumption of Mediterranean citizens was estimated using the FAOSTAT element “protein supply quantity” (code 674) (g protein per capita d^−1^). Equation 5 was used to estimate the *average per capita daily consumption of bone free meat* (C_BFM_) (g capita^−1^ d^−1^) *in 7MED* for each meat type (beef, mutton, pork and poultry), to avoid biases due to disproportional population size and meat consumption among the studied countries.

5 $$C_{BFM} = \left[ {\sum\limits_{1}^{n} {\left( {\frac{{ITEM_{Pi} }}{{Prt_{frac} }} \times Population_{i} } \right)} } \right]/\sum\limits_{1}^{n} {Population_{i} }$$

where *n* represents the 7MED countries, *ITEM*_*Pi*_ is the daily protein supply quantity for the specific meat type *P* in country *i*; *Prt*_*frac*_ is the fraction of protein content of the meat, calculated considering different meat cuts^23^, with *Prt*_*frac*_ values of 0.21 for beef meat (anterior and posterior cuts), 0.21 for mutton, 0.19 for chicken (with skin), 0.17 pork (average of steak, sausages, ham); *Population*_*i*_ is the number of individuals present in the country *i*. All data refers to the year 2017. The weekly consumption was estimated as C_BFM_ multiplied by 7 (days in a week).

The weekly food deviation of the 7MED from the ideal MD pattern was calculated for each of the 15 food groups as the difference between the amount of weekly food intake proposed by the MD (average value of the 3 scenarios of Table 1) and the calculated weekly consumption data (with 2017 as the reference year). The weekly deviation expressed in grams was multiplied by the weighted CF factors of each food group to derive total CO_2_eq emissions (Supplementary Table 4). The total CO_2_eq daily net excess was estimated by summing the CO_2_eq weekly deviations of the 15 groups divided by 7 (days in a week). For the meat category, the excess of each meat type (beef, mutton, pork and poultry) was calculated separately and multiplied by the respective CF value, and then the emissions were summed again to represent red meat (beef, mutton and pork) and poultry separately.

Kcal deviation per week (Supplementary Table 4) per food group was also derived by multiplying the weekly deviation in grams of the food group by its weighted kcal value. The latter was calculated from the kcal values of the single food items belonging to the group, with a similar approach to Eq. 3.

### Annual per capita E_GHG_ from meat consumption of EU citizens between 1961 and 2017

The E_GHG_ associated with apparent meat consumption in the EU from 1961 to 2017 was estimated based on the element “food” (code 5142) in the FAOSTAT food balance database. To analyse the meat consumption in the 28 countries investigated in this study for a timeframe when EU 28 was not existing yet, we listed the countries present in the EU 28 in 2017 and then we analysed the trend of each country from 1961. Due to different political assets, Czechoslovakia data was considered from 1961 to 1992, and Belgium-Luxembourg data from 1961 to 1999. Croatia data is missing before 1991, which might have resulted in some bias between the data of meat consumption in 7MED before and after 1991. However, the estimate of emissions from meat consumption represents a weighted average and not the sum of total emissions, which means that any error would be considered only minor. To estimate the CO_2_eq emissions for each meat type, we used an average CF value based on bone free meat and meat with bone CF data^14,15^.

## Results and discussion

To meet the 2050 global emission reduction target, the EAT-Lancet Commission has proposed a planetary health dietary pattern formulated to represent a boundary of 5 Gt of CO_2_eq per year (uncertainty range of 4.7–5.4 Gt CO_2_eq yr^−1^) from food production, about half of the total GHG emissions (CO_2_eq) expected in 2050 with the RCP2.6 scenario (2 °C temperature rise)^2^. The CFs values used in this study to estimate the MD E_GHG_ were applied to the recommended average weekly per capita food consumption rates reported by Willet et al.^2^, obtaining a value of 2.49 kg CO_2_eq capita^−1^ d^−1^, which we used as a reference value to assess the climate sustainability of the MD. We then assessed that adherence of an average citizen to the MD pattern, as reported in Table 1, would lead to a daily per capita E_GHG_ of 2.31 ± 0.14 kg CO_2_eq capita^−1^ d^−1^. This value is in agreement with the estimated E_GHG_ impact of the EAT-Lancet dietary recommendations^2^, corroborating the important role that the MD could play in contributing to the EU E_GHG_ mitigation targets.

**Figure 1:**
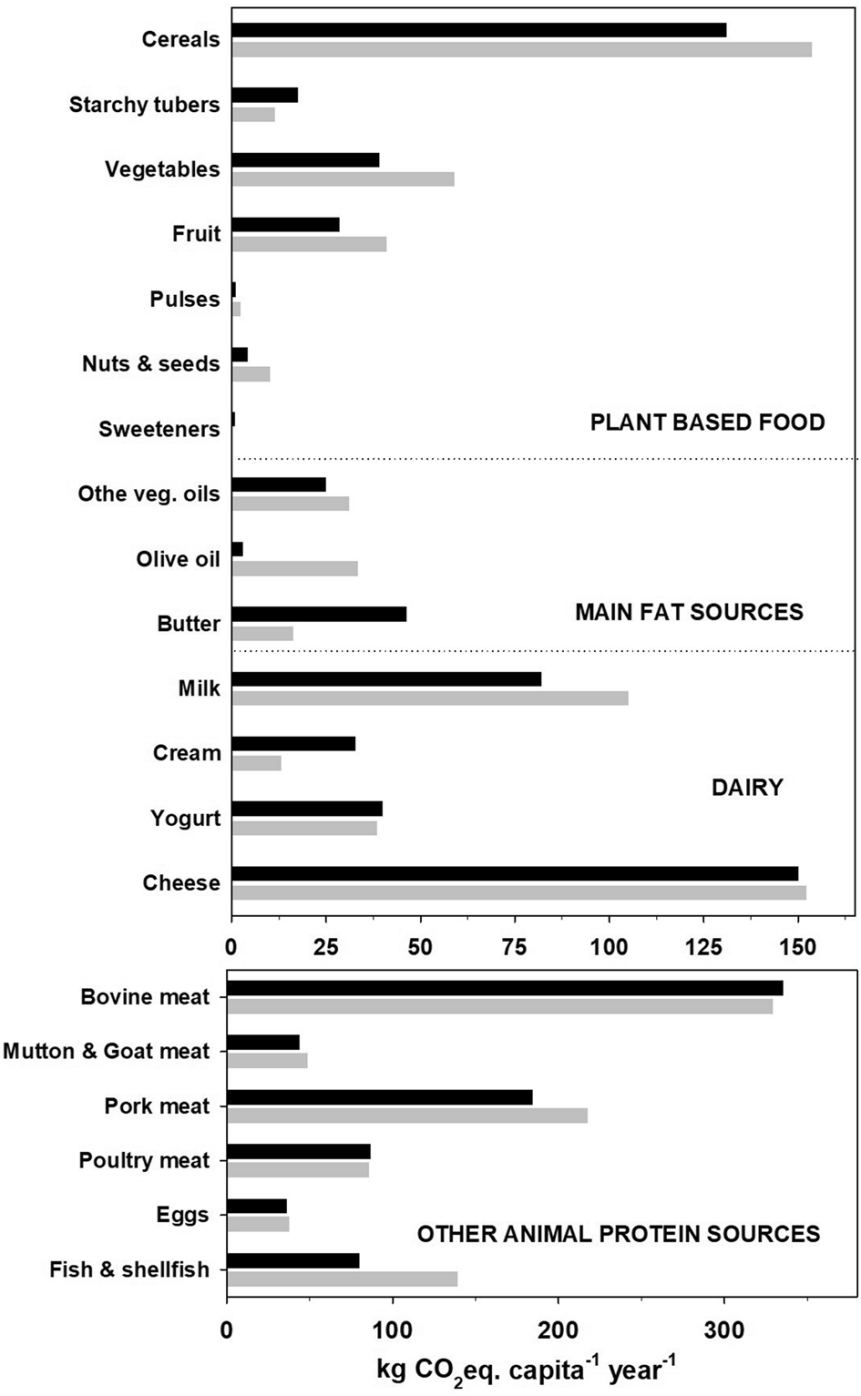
Annual per capita E_GHG_ (kg CO_2_eq. capita^−1^ yr^−1^) associated with food consumption of EU citizens. E_GHG_ of food groups refer to the weighted mean food consumption of Mediterranean citizen (7MED, grey bars) and other EU countries (21OTHER, black bars) in 2017.

In contrast, our analysis of actual food consumption patterns in 7MED shows that Mediterranean citizens were far from this ideal dietary target emitting, on average, about 4.46 kg CO_2_eq capita^−1^ d^−1^, which is almost double the expected daily E_GHG_ from the ideal MD pattern. Moreover, average daily E_GHG_ in 7MED was comparable to that of the other EU citizens (21OTHER), which is 4.03 kg CO_2_eq capita^−1^ d^−1^ (Fig. 1). An in-depth analysis of E_GHG_ associated with the dietary consumption patterns of the two groups (Fig. 1) shows that there are no major differences in terms of the highest-emitting food: beef meat, mutton meat and cheese^2,14^. While E_GHG_ from poultry consumption were comparable, the 7MED countries showed higher E_GHG_ from pork and fish consumption. E_GHG_ from added fat sources were comparable, as 21OTHER citizens consumed slightly more than twice the amount of butter (CF 8.48 kg CO_2_eq kg^−^1) used in Mediterranean countries, while 7MED citizens consumed 8 times more olive oil (CF of 3.26 kg CO_2_eq kg^−1^), which levelled off the difference. Both groups had a comparable use of seed oil. + 7MED citizens tended to consume more plant-based food, which is one of the three pillars of the MD^19^. Notably, the difference in E_GHG_ between the two groups was relatively small (Fig. 1), as the CF magnitude of plant-based food is significantly lower than that of animal-based food^2,14,15,26^.

**Figure 2:**
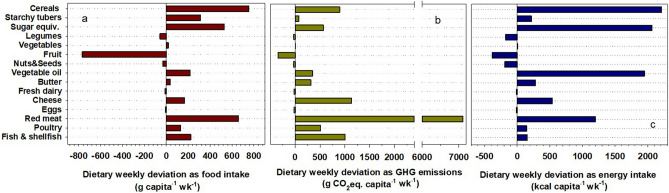
Deviations of food consumption patterns of Mediterranean citizens (7MED) from the reference MD. Data are represented as total weekly deviation of each analysed food group to take into account a complete dietary plan with specific weekly frequency consumption of different food items. Differences are reported as (**a**) fresh food weight intake (g), (**b**) E_GHG_ derived from C footprint of consumed food items, (**c**) calorie intake. Positive values indicate an extra weekly intake, negative sign a deficit weekly intake.

**Figure 3:**
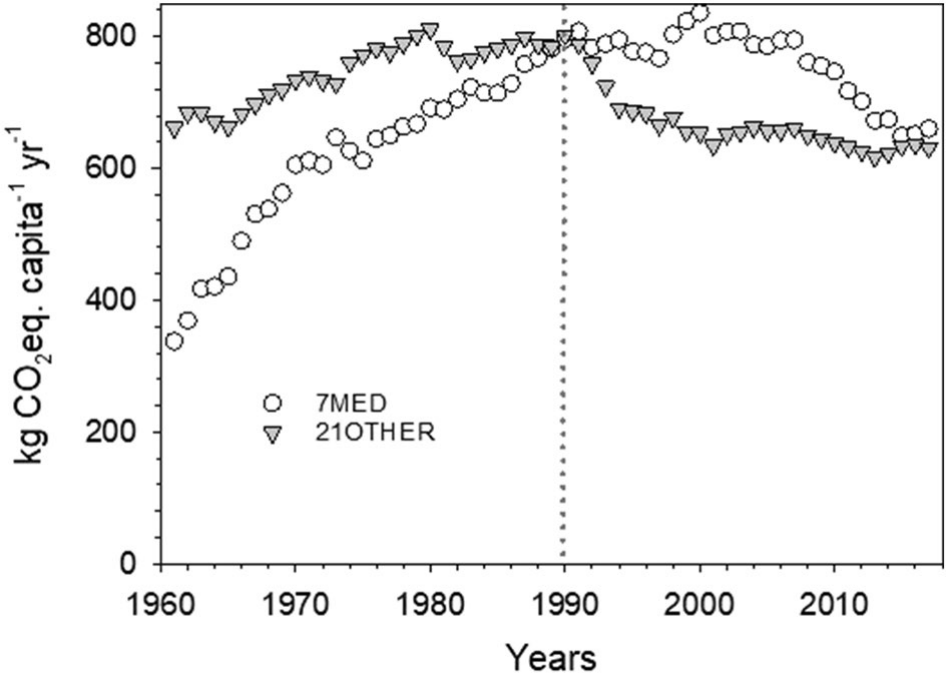
Annual per capita GHG emissions associated to meat consumption in European countries from 1961 to 2017. Total per capita yearly E_GHG_ (kg CO2 eq. capita^−1^ yr^−1^) associated to apparent consumption of beef, mutton and goat, pork and poultry meat by citizen in 7MED (white circles) and 21OTHER (grey triangles) countries from 1961 to 2017.

**Figure 4:**
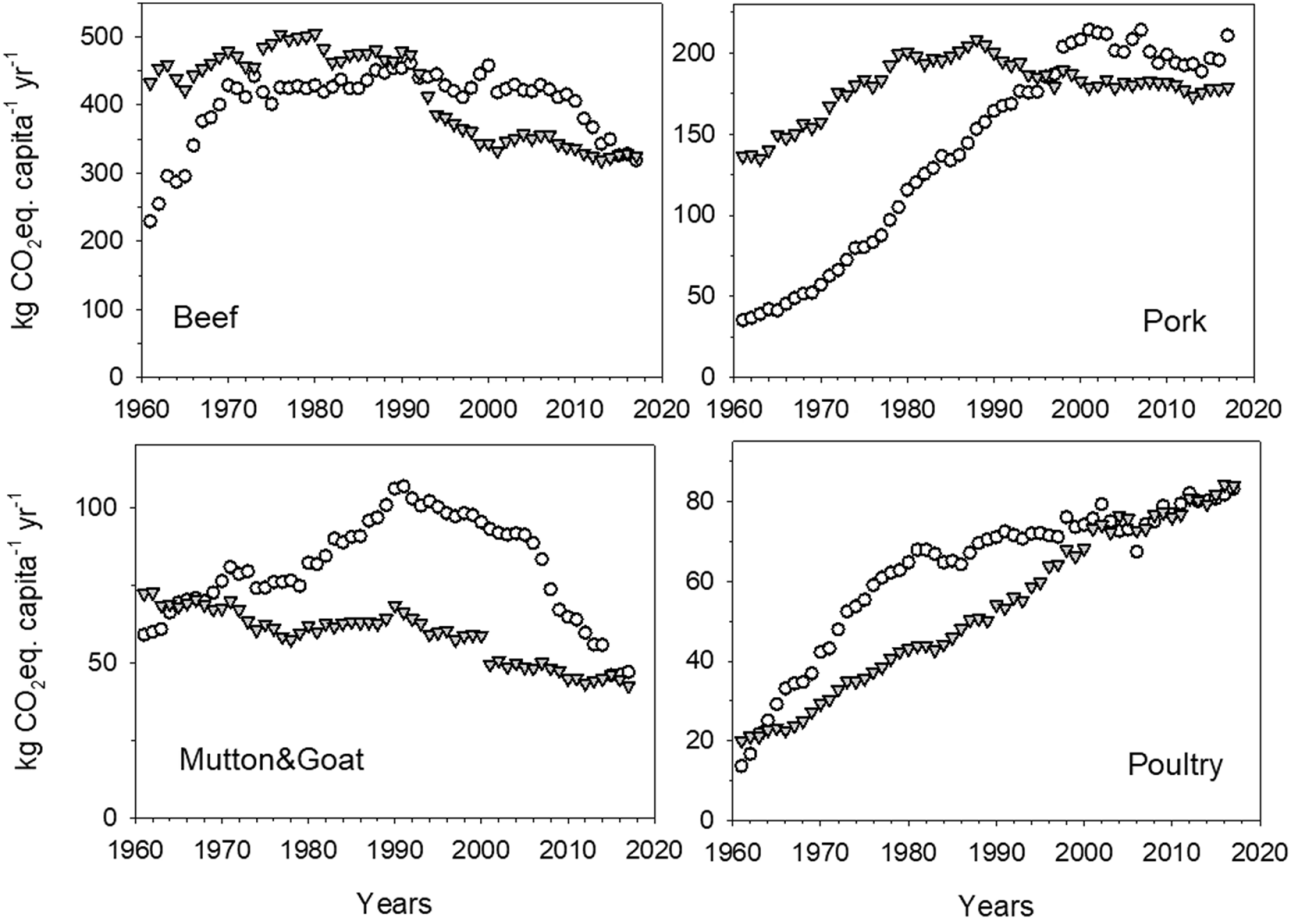
Annual per capita GHG emissions associated to beef, pork, mutton & goat and poultry meat consumption in European countries from 1961 to 2017. Total per capita yearly E_GHG_ (kg CO_2_ eq. capita^−1^ yr^−1^) associated to apparent consumption of each meat type (beef, mutton and goat, pork and poultry) by citizen in 7MED (white circles) and 21OTHER (grey triangles) countries from 1961 to 2017.

To better comprehend the current patterns of food consumption and related GHG impacts in Mediterranean countries, we compared the ideal weekly MD plan with the real weekly food consumption pattern of Mediterranean EU citizens. Our results show a clear deviation of real dietary habits from the ideal MD pattern. The per capita dietary intake exceeded the energy and nutrient requirements of the reference MD diet, which was largely the result of increased intake of meat, mostly red meat, cheese, additional fats and carbohydrate-rich food (Fig. 2a, c). Food and calorie excess not only have major consequences on healthy perspective^2,9^ but also result in a considerable daily excess of E_GHG_, equal to 1.8 kg of CO_2_eq capita^−1^ d^−1^ (Fig. 2b). Whereas carbohydrate-rich food and additional fats significantly contributed to the calorie excess (Fig. 2c, Supplementary Table 4, 2 daily extra portions of grains, 1 extra portion of potatoes, 27 of sugars equivalents, 5 daily extra portions of oil and 37 extra grams of butter per week), the biggest contribution to the E_GHG_ excess came from over consumption of animal proteins (Fig. 2b, Supplementary Table 4, 2 portions of beef meat, 5 of pig meat, 1½ of poultry meat, 2 of cheese per week). Overall, the animal protein excess was 70% of the estimated total E_GHG_ excess of 1.8 kg of CO_2_eq capita^−1^ d^−1^, with red meat alone representing 56%. A low intake of red meat has been associated with traditional Mediterranean diets and is considered one of the main reasons for Mediterranean people’s longevity. A low incidence of coronary heart disease and mortality was identified in the island of Crete, Greece, in the 1960s. This was associated^27^ with an average intake of red meat and poultry that combined was only 35 g capita^-1^ d^-1^. We calculated that, in Mediterranean countries in the 1960s, the total apparent meat consumption (FAOSTAT definition which includes meat with bone for some categories of cuts and animals), was around 25 kg capita^-1^ yr^−1^, corresponding to 68 g capita^−1^ d^−1^, which is in agreement with the daily intake of edible meat cut estimated in Crete by Kromhout et al.^27^. In 2017 the total apparent meat consumption of Mediterranean citizens had reached 86 kg capita^−1^ yr^−1^ (around 235 g capita^−1^ d^−1^), more than three times the meat consumption estimated in 1960 (Supplementary Fig. 1). This increase has coincided with a dramatic increase in E_GHG_ (Fig. 3). In the early 1990s, per capita E_GHG_ from meat consumption in Mediterranean countries were already above the E_GHG_ values estimated for the other EU countries, and only recently have the two groups started to converge, largely as a result of reduced beef consumption (Supplementary Fig. 1, Fig. 4).

It is important to note that the estimates of E_GHG_ provided in this study are mainly based on an analysis of minimally processed food items and exclude highly processed food and beverages. While this allowed a direct comparison with the MD, which is traditionally based on fresh and locally produced food ^11^ and excludes many common beverages, it might introduce some underestimation of the total food-related E_GHG_ of EU citizens. In this respect, adherence to MD diet would be even more beneficial to climate targets than estimated, as fresh local and traditional food forms the base of Mediterranean food preparation. With an excess of 1.8 kg CO_2_eq capita^−1^ d^−1^ applied to the population of the 7MED countries, the potential total savings from adhering to MD recommendations would be approximately 105 Mt CO_2_eq y^−1^.

## Conclusions

The results of this study support the positive role that the MD could have on EU climate mitigation targets if it were fully adopted by Mediterranean citizens. The analysis also shows that the nutritional transition experienced by Mediterranean countries, in particular over the last 30 years, has undermined this potential. A significant dietary shift to the dietary patterns of the Mediterranean tradition would bring significant environmental gains as well as increased health benefits related to non-communicable diseases, including a lower incidence of cancer, cognitive disease and cardiovascular diseases as well as for metabolic syndrome, obesity and type 2 diabetes^2,25^. As indicated by Willet et al. ^2^ “the absence of scientific targets for achieving healthy diets from sustainable food systems has been hindering large-scale and coordinated efforts to transform the global food system”. The present analysis provides guidance to effectively implement actions and measures to support sustainable and healthy dietary shifts at the Mediterranean and EU levels.

## Supplementary Information

Supplementary Information.
