# Supplementary material for: The positive climate impact of the Mediterranean diet and current divergence of Mediterranean countries towards less climate sustainable food consumption patterns
Source: Sci Rep. 2022 May 25;12:8847. doi: 10.1038/s41598-022-12916-9 (PMC9132980; doi:10.1038/s41598-022-12916-9)
Supplement: Supplementary file 1 — Supplementary Information. [file 41598_2022_12916_MOESM1_ESM.pdf]

SUPPLEMENTARY INFORMATION

The positive climate impact of the Mediterranean diet and current divergence of Mediterranean countries towards less climate sustainable food consumption patterns

Simona Castaldi[a*], Katarzyna Dembska[b], Marta Antonelli[b,c], Tashina Petersson[d], Maria Grazia Piccolo[a], Riccardo Valentini[c,d,e]

Affiliations

[a]Department of Environmental, Biological and Pharmaceutical Science and Technology, University of Campania "Luigi Vanvitelli", via Vivaldi 43, 81100 Caserta, Italy, simona.castaldi@unicampania.it, mariagrazia.piccolo@studenti.unicampania.it

[b]Barilla Center for Food & Nutrition Foundation, via Madre Teresa di Calcutta, 3/a, Parma, Italy, katarzyna.dembska@barillacfn.com, marta.antonelli@barillacfn.com

[c]Euro-Mediterranean Centre on Climate Change, via Augusto Imperatore 16, 73100, Lecce, Italy.

[d]Department for Innovation in Biological, Agro-food and Forestry Systems, University of Tuscia, via Camillo de Lellis 4, 01100 Viterbo, Italy, tashina.petersson@unitus.it, rik@unitus.it

[e] Department of Ecology, Russian Timiryazev State Agrarian University, Timiryazevskaya st., 49, 127550 Moscow, Russia

***Corresponding author:** simona.castaldi@unicampania.it

Supplementary Table 1 – *Data used to estimate the greenhouse gas emissions (E_{GHG}) associated to apparent food consumption in EU.* Columns report the following data: plant based food, meat and fish FAOSTAT ITEM code, FAOSTAT ITEM name and year of reference used to extract "food"data (FAOSTAT "element" code 5142) from the FAOSTAT food balance database https://www.fao.org/faostat/en/#data/FBS, for the countries of EU in 2017; the carbon footprint (CF) values used to attribute greenhouse gas emissions (E_{GHG}) to the FAOSTAT food items. The latter were extracted from the Su-Eatable LIFE database by Petersson et al.[15] and the specification of the used item, typology or sub-typology CF value, associated to the FAOSTAT item name, is reported in the last column.

**FAOSTAT Item Code**	**FAOSTAT Item name**	**Year**	**C footprint value (CF)** kg CO_2eq. kg^{-1} or l^{-1}	**Reference item/typology name as reported in the database by Petersson et al.[15]**
2511	Wheat and products	2017	1.035	Average: pasta/bread (1:1)
2513	Barley and products	2017	0.86	Bread (typology)
2514	Maize and products	2017	0.86	Bread (typology)
2515	Rye and products	2017	0.86	Bread (typology)
2516	Oats	2017	0.86	Bread (typology)
2517	Millet and products	2017	0.86	Bread (typology)
2520	Cereals, Other	2017	0.86	Bread (typology)
2531	Potatoes and products	2017	0.24	Potato (item)
2532	Cassava and products	2017	0.27	Starchy tubers (typology)
2533	Sweet potatoes	2017	0,27	Starchy tubers (typology)
2534	Roots, Other	2017	0.27	Starchy tubers (typology)
2535	Yams	2017	0.27	Starchy tubers (typology)
2536	Sugar cane	2017	-	
2537	Sugar beet	2017	-	
2542	Sugar (Raw	2017	0.78	Sugar (typology)
2543	Sweeteners, Other	2017	0.78	Sugar (typology)
2546	Beans	2017	0.43	Bean (item)
2547	Peas	2017	0.52	Legumes (typology)
2549	Pulses, Other and prod.	2017	0.52	Legumes (typology)
2555	Soyabeans	2017	0.56	Soybean (item)
2551	Nuts and products	2017	1.11	Nuts (typology)
2556	Groundnuts (Shelled	2017	1.11	Nuts (typology)
2557	Sunflower seed	2017	0.88	Seeds (typology)
2558	Rape and Mustardseed	2017	0.88	Seeds (typology)
2559	Cottonseed	2017	0.88	Seeds (typology)
2560	Coconuts - Incl Copra	2017	0.88	Seeds (typology)
2561	Sesame seed	2017	0.88	Seeds (typology)
2562	Palm kernels	2017	0.88	Seeds (typology)
2563	Olives (incl. preserved)	2017	0.64	Olives (item)
2570	Oilcrops, Other	2017	1.98	Average (palm oil, peanut oil, rapeseed oil, soybean oil, sunflower
2571	Soyabean Oil	2017	1.98	As above
2572	Groundnut Oil	2017	1,98	As above
2573	Sunflowerseed Oil	2017	1.98	As above
2574	Rape and Mustard Oil	2017	1.98	As above
2575	Cottonseed Oil	2017	1.98	As above
2576	Palmkernel Oil	2017	1.98	As above
2577	Palm Oil	2017	1.98	As above
2578	Coconut Oil	2017	1.98	As above
2579	Sesameseed Oil	2017	1.98	As above
2582	Maize Germ Oil	2017	1.98	As above

2586	Oilcrops Oil, Other	2017	1.98	As above
2580	Olive Oil	2017	3.27	Olive oil (item)
2601	Tomatoes and products	2017	0.45	Tomato (item)
2602	Onions	2017	0.22	Onion (item)
2605	Vegetables, Other	2017	0.33	Vegetables openfield (typology)
2611	Oranges, Mandarines	2017	0.30	Orange (item)
2612	Lemons, Limes and	2017	0.22	Lemon (item)
2613	Grapefruit and products	2017	0.34	Citrus fruit (sub-typology)
2614	Citrus, Other	2017	0.34	Citrus fruit (sub-typology)
2615	Bananas	2017	0.82	Banana imported (item)
2616	Plantains	2017	0.82	Banana imported (item)
2617	Apples and products	2017	0.25	Apple (item)
2618	Pineapples and	2017	0.55	Pineapple imported (item)
2619	Dates	2017	0.60	Date (item)
2620	Grapes and prod. (excl	2017	0.31	Grapes (item)
2625	Fruits, Other	2017	0.40	Fruit open field (typology)
2630	Coffee and products	2017	6.71	Ccoffee ground & parchment
2640	Pepper	2017	0.84	Spices (typology)
2641	Pimento	2017	0.84	Spices (typology)
2645	Spices, Other	2017	0.84	Spices (typology)
2745	Honey	2017	1.74	Honey (item)
2805	Rice and products	2017	2.19	Rice (item/typology)
2731	Bovine Meat	2017	21.85	Average (beef bone free meat, beef meat with bone; typologies)
2732	Mutton & Goat Meat	2017	22.61	Average (lamb bone free meat, lamb meat with bone; typologies)
2733	Pigmeat	2017	4.58	Average (pork bone free meat, pork meat with bone; typologies)
2734	Poultry Meat	2017	3.01	Average (poultry bone free meat, poultry with bone; typologies)
2735	Meat, Other	2017	13.01	Average*
2736	Offals, Edible	2017	-	**
2744	Eggs	2017	3.2	Eggs (item)
2761	Freshwater Fish	2017	5.19	Fish (typology)
2762	Demersal Fish	2017	5.19	Fish (typology)
2763	Pelagic Fish	2017	5.19	Fish (typology)
2764	Marine Fish, Other	2017	5.19	Fish (typology)
2765	Crustaceans	2017	7.04	Crustacean (sub-typology)
2766	Cephalopods	2017	5.80	Cephalopods (sub-typology)
2767	Molluscs, Other	2017	0.46	Bivalves (sub-typology)

*For the item "Meat, Other" (code 2735) an average of the all the available meat data was used. **The tons of the item "Offals, Edible" (FAOSTAT code 2736) were summed up to the tons of the four main meat items "Bovine Meat", "Mutton & Goat Meat", "Pigmeat", "Poultry Meat" in proportion to the normalized fraction of each meat item consumed in EU in 2017, as no specific CF is available for this item.

Supplementary Table 2 - Aggregated category definition used to attribute CF values, extracted from Petersson et al.[15], to dairy products.

	**C footprint value (CF)** Kg CO_2eq. kg^{-1} or l^{-1}	**Reference item/typology name as reported in the database by Petersson et al.[15]**
Milk	1.44	Milk (typology, animal)
Cream	5.45	Cream (item, animal)
Yogurt	2.55	Yogurt (typology, animal)
Butter	8.48	Butter (item/typology)
Fresh cheese	5.45	Cheese fresh (typology)
Hard and semi-hard cheese	9.59	Cheese hard & semi hard (typology)

Supplementary Table 3 – *Tons of dairy products consumed in EU28 in 2017* considering production minus export plus import as reported in the balance sheets "Dairy by Country" in Clal.it https://www.clal.it/en/?section=produzioni. The fraction of fresh cheese (cod. Eurostat D7116) over total is 35%, based on an EU average, as reported in Clal.it. Data are grouped following the classification reported in Supplementary Table 2.

Dairy group	EU-28 Tons	21 OTHER Tons	7 MED Tons	Cipro Tons	Croatia Tons	Greece Tons	Italy Tons	Malta Tons	Portugal Tons	Spain Tons
Milk[a]	31,232,000	21,432,200	9,799,800	98,500	388,600	739,900	4,045,600	59,800	752,300	3,715,100
Cream[b]	2,594,000	2,271,500	322,500	3,900	33,700	41,000	190,900	0	22,000	31,000
Yogurt[c]	7,947,600	5,924,000	2,023,600	9,200	82,500	61,000	572,000	2,900	214,000	1,082,000
Butter[d]	2,315,000	2,056,200	258,800	2,000	7,600	11,900	143,900	600	24,800	68,000
Cheese[e]	9,401,000	6,909,300	2,491,700	15,000	55,300	273,000	1,356,000	7,700	123,700	661,000

a includes production of drinking milk (Cod. Eurostat D2100), concentrated milk (Cod. Eurostat D3200), WMP (Cod. Eurostat D3100_X_3113), Partially Skimmed Milk Powder (Cod. Eurostat D3112), SMP (Cod. Eurostat D3113), buttermilk powder (Cod. Eurostat D3130), drinks with a milk base (Cod. Eurostat D9100), export and import data with Harmonized System Code 190190 (FFMP), 040210 (SMP), 04011010+04012011+04012091 (processed milk), 190110 (Preparations for infant use), 040221+040229 (WMP), 040291+040299 (condensed milk); **b** includes production of cream for direct consumption Cod. Eurostat D2200V, export and import data with Harmonized System Code 040130+040140+040150; **c** production of acidified milk (yoghurts and other) (Cod. Eurostat D4100); **d** includes production of butter (Cod. Eurostat D6000), rendered butter and butteroil (Cod. Eurostat D6200), other yellow fat dairy products (Cod. Eurostat D6900), export and import of data with Harmonized System Code 0405; **e** includes production data of cheese (Cod. Eurostat D7100), export and import data with Harmonized System Code 0406.

Reference data for Eurostat codes[27] https://ec.europa.eu/info/sites/default/files/food-farming-fisheries/farming/documents/eu-dairy-historical-production-stocks-series_en.pdf. World dairy trade data (Harmonized System Code) on Clal's database Dairy World Trade (DWT) come from IHS/GTA and are processed by Clal.it.

Supplementary Table 4 – Excess of per capita food consumption, expressed as grams week^{-1}, kcal week^{-1}, CO_2equ. emissions week^{-1}, calculated for the population of 7MED countries, by comparing their weekly food consumption data for 2017 with the average ideal weekly MD consumption of food groups reported in Table 1. The Mediterranean Pyramid Diet (MPD) frequency indications, daily portion (g) and weekly frequency, refer to data reported in Table 1 (see also the section of methods for a detailed explanation of assumptions). The average weekly input is calculated as the mean of the 3 weekly input scenarios reported in Table 1.

					**Per capita excess**		
**Food type**	**MPD frequency indications**	**Daily portion (g)**	**Weekly frequency**	**Average weekly input (g)**	**g wk^{-1}**	**kcal wk^{-1}**	**kg CO_2eq. wk^{-1}**
Cereals	1-2/meal	300	7	1900	753.1	2210.4	896.2
Legumes	>2/ week	50	2-4	133	-57.1	-178.2	-28.0
Potatoes	<3/ week	300	2	600	312.7	218.9	75.0
Vegetables	>2/meal	400	7	2800	18.3	7.3	7.5
Fruit	1-2/meal	300	7	3150	-767.0	-383.5	-345.1
Nuts&seeds	1-2/day	30	7	210	-30.1	-189.4	-33.1
Red meat	<2/week	100*	1	100	658.9	1198.6	7113.2
Poultry	2/week	100	2-3	233	131.2	144.4	509.2
Cheese	NS	50-100**	1-3	192	165.7	538.4	1131.5
Dairy	2/day	300	7	1750	-11.4	-7.5	-23.4
Butter	0	0	0	0	37.1	281.5	314.1
Eggs	2-4/week	100	2-3	200	-7.2	-9.2	-22.9
Vegetable	every meal	40	7	280	216.4	1947.7	348.4
Fish	>2/week	150	2-4	450	222.9	156.0	1008.3
Sugar equ.	NS	20	7	140	529.5	2064.9	566.2

*Includes beef, lamb and pork meat, 100 g of each meat type being alternative in the week dietary plan and not additional; ** portions of cheese are considered as 100g of fresh cheese or 50 g of hard/semi hard cheese; NS not specified

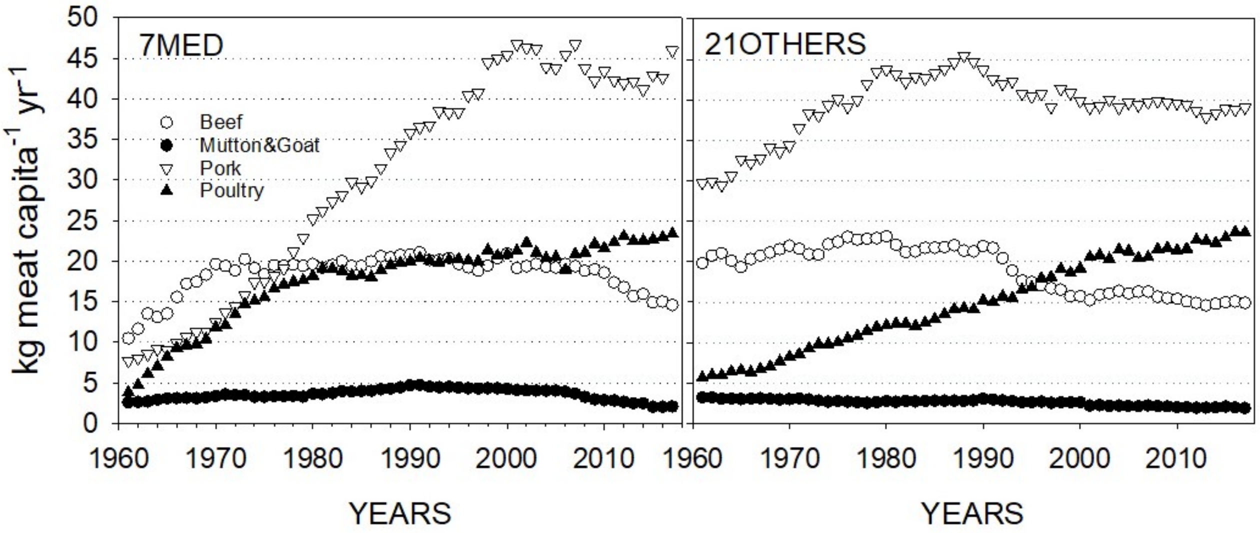

Supplementary Fig. 1 - Trends of apparent meat consumption per capita per year estimated over the period 1961-2017 for the 7MED countries and 21OTHER countries from FAOSTAT data 2017 for EU28 (see methods for a detailed explanation of FAOSTAT data calculations over the 1961-2017 period).
